# Performance of EuroSCORE II in the prediction of in-hospital death after on pump versus off pump CABG

**DOI:** 10.1186/1749-8090-8-S1-O204

**Published:** 2013-09-11

**Authors:** M Sales, E Lúcio, J Frota Filho, A Rösler, J Fraportti, G Constantin, P Leães, V Lima, M Pontes, F Lucchese

**Affiliations:** 1 Cardiovascular Surgery, Hospital São Francisco, Porto Alegre, Brazil

## Background

The role of EuroSCORE II (ES II) in risk prediction after on pump CABG X off pump (OPCAB) is unknown. Our aim was to evaluate and compare predictive power of ES II on pump X OPCAB.

## Methods

All consecutive pts for CABG (Jan 2010-Dec 2012). Choice of technique was based on anatomic and clinical variables. We evaluated demographic, clinical, operative variables, ES and in-hospital outcomes. Ability of ES II was tested for performance (observed/expected [O/E] mortality ratio) and accuracy (area under the ROC curve, AUC).

## Results

862 pts (63±10y, 69% male); 57% OPCAB. Median ES II=1,12%; ES I=2,45%. Observed mortality was 2,9%. OPCAB pts had lower glucose, higher ejection fraction, less previous surgeries, less LMCA lesions, smaller bleeding and less distal anastomosis (3,2±1,2 x 3,8±1,0, p<0,001). They had similar use of LITA (89,8% x 86,2%, p=0,102) and complete revascularization (94,9% x 94,5%, p=0,820). In bivariate analysis, OPCAB had lower in-hospital mortality (1,8% x 4,3%, p=0,031) and PO bleeding (1,0% x 3,0%, p=0,035), and more new revascularization (1,8% x 0%, p=0,006). Incidence of MACCE and stroke was similar. In multivariate analysis, independent predictors of death were on pump CABG (OR=3,08 [1,22-7,80] p=0,017) and ES II (OR=1,29 [1,04-1,60] p=0,023). Performance (O/E ratio) of ES II was moderate in all cohort (O/E 1,75, IC95 1,46-3,71), very good for OPCAB (O/E 1,11, IC95 1,03-1,22 ), and poor for on pump CABG (O/E 2,51, IC95 2,31-2,79), p<0,05. Accuracy: In all cohort, ES II showed fair accuracy (AUC 0,725), better than ES I (AUC 0,683). In OPCAB, accuracy was moderate (AUC 0,681), much better than ES I (AUC 0,571). In the on pump CABG, ES I and ES II showed fair accuracy (AUC 0,743 e 0,746, respectively).

## Conclusions

EuroSCORE II showed moderate to good accuracy in all surgical groups. It has a better predictive ability in OPCAB than in on pump CABG. Predictive ability of ES II was better than ES I in all CABG patients.

